# Learning in intensive care during the COVID-19 pandemic postgraduate critical care nursing students’ experiences

**DOI:** 10.5116/ijme.6399.ea3f

**Published:** 2022-12-29

**Authors:** Angelica Fredholm, Åsa Engströlm, Maria Andersson, Anna Nordin, Mona Persenius

**Affiliations:** 1 Centre for Clinical Research, County Council of Värmland, Karlstad, Sweden; 2 Department of Health, Education and Technology, Division of Nursing and Medical Technology, Lulea University of Technology, Lulea, Sweden; 3 Faculty of Health, Science, and Technology, Department of Health Science, Karlstad University, Karlstad, Sweden

**Keywords:** COVID-19, critical care nursing, critical care nursing students, preceptorship, authenticity, autonomy, attachment, professional development

## Abstract

**Objectives:**

This study explored postgraduate critical care nursing students’ experiences of learning in the ICU during the COVID-19 pandemic and to understand these experiences in relation to self-directed learning and professional development.

**Methods:**

An explorative qualitative design was used. Eight postgraduate critical care nursing students from two different universities were interviewed. Questions focused on learning, supervision, ethically difficult situations, issues regarding communication, as well as the impact of the pandemic on students’ health. Interviews thematically analyzed, and further analyzed using a theoretical framework focusing self-directed learning and professional development containing the concepts of autonomy, authenticity, and attachment.

**Results:**

The result consists of three themes: 1) Attachment with subthemes Attachment to the patient, Attachment to family and friends, Attachment to the ICU-context, and Attachment to the clinical supervisor.  2) Authenticity with subthemes Experiencing a varying degree of authenticity, Clinical reasoning about how to prioritize care. 3) Autonomy with subthemes Being just a student – with limited responsibility, taking responsibility, and having worries regarding one’s professional development. Conclusion: Findings show the need for participation in the ICU community of practice without the demands and responsibility of full participation. Students need to be given the opportunity to form a relationship with practice. For attachment, participation, and consequently professional development to take place, there is need for inviting students to be a part of the team even during such straining circumstances as an ongoing pandemic. These findings can advance the understanding of how to organize clinical education during future crisis such as a new pandemic.

## Introduction

One challenge for clinical education is for clinical supervisors to find a balance between furthering students’ independence and professional development and at the same time guaranteeing patient safety.^1^ During the COVID-19 pandemic, clinical education has changed for supervisors and postgraduate nursing students within the ICU context. The normal situation with few clinical supervisors has changed, supervisors have a larger workload and must at the same time also introduce and supervise new co-workers. This might have had an impact on the supervision and on self-directed learning and professional development.

There has been a rapid acceleration in levels of responsibility for ICU nurses during the pandemic because of the expansion of their professional role, resulting in a significant increase in their regular workload^2^^,^^3^ with a significantly higher number of patients per nurse and a significantly higher nursing activity score per nurse during COVID-19.^4^

Clinical education is a complex phenomenon that can be described in terms of e.g., workplace learning, situated learning, and experience-based learning. Common to these perspectives is the combination of theory and practice and the social nature of learning where interaction with others is fundamental.^5^

Experiences gained in the "messiness and complexity of practice” cannot be reproduced elsewhere.^6^  Independence in learning and autonomy are connected to factors such as motivation, control, ability to seek and apply knowledge, critical thinking, identity, learning needs, and ability to evaluate learning outcomes, and then make independent choices.^7^^-^^13^ Based on research regarding self-directed learning and professional development^14^^-^^16^ the concepts of attachment, authenticity, and autonomy are identified as important to support student learning in the clinic.

Clinical educators or supervisors must combine their clinical work with education and supervision, and thus focus their attention on the individual student’s learning, while also handling their own interaction with patients.^18^ The sociocultural learning environment has an impact on how students can make use of their clinical education.^5^ How students perceive their connection to the supervisor is vital for how they perceive their clinical education and for^14^^,^^16^ learning and professional development.

Due to the extraordinary pressure and shortage of critical care nurses (CCN:s), several ICUs needed to change their competence structure and include nurses from other specialities and nurses without postgraduate training (general care nurses) in the ICU team.^17^ Even professionals who do not usually work in health care could receive a crash course to be able to work as assistants in the ICU.^19^ Critical care nurses were thus introducing, supervising, and leading a team of nurses without specific ICU competences, at the same time as they were also in charge of patient care^3^^,^^20^ and supervising nursing students and postgraduate critical care nursing students. The CCN:s experienced challenges working with new co-workers and teams, and they were struggling with a lack of defined roles and maintaining existing working relationship.^21^ Patient safety and quality of care were compromised, and nursing care was severely deprioritized.^17^^,^^20^ All this created additional cognitive and emotional demands for the nurses.^3^ Worldwide, nursing unions have announced that many CCN:s have reported COVID-related occupational diseases, been on sick leave or chosen to resign after a period with high workload and a poor work environment. The shortage of CCN:s, as well as preceptors with experience and confidence in precepting, has further complicated the post-graduate education.^22^

Due to the demands created by the COVID-19 pandemic, there are reasons to assume that the complex phenomenon that is clinical education has been affected regarding supervision, self-directed learning, and professional development. During the pandemic considerable strain has been put on the intensive care. Therefore, the aim was to explore postgraduate critical care nursing students’ experiences of learning in the ICU during the COVID-19 pandemic and to understand these experiences in relation to self-directed learning and professional development.

## Methods

The conducted research is methodologically positioned within the interpretative qualitative research paradigm. Phenomena under study a were explored and interpreted in order to capture students’ first-hand experience of the studied phenomena. As such, data was interpreted openly and inductively to explore the experiences. Later in the analysis theory was used to further interpret, deepen, and illuminate findings.

### Context and participants

A convenience sample with participants with first-hand experience of the phenomena under study was used. Study participants consisted of postgraduate critical care nursing students (students) in the one-year educational program at advanced level “Study Program for Specialist Nursing in Intensive Care” at two universities in central and northern Sweden. Both universities had local regulations with their respective health care organizations regarding supervision and critical care nurses providing supervision. The program led to a master’s degree in critical care nursing.

While the students in the educational program starting in autumn 2019 performed their 12 weeks of clinical education in the spring of 2020, in the midst of the pandemic outbreak, the students in the program starting in autumn 2020 performed their 12 weeks of clinical education in the spring of 2021. The length of each clinical education varied between four to six weeks.

All students were women, ages 26 to 35 years old, and had work experience as Registered Nurses of four to ten years. In addition, one had another specialist nursing degree and four of them had experience of working as enrolled nurses. All but one had a trainee position, five had worked at their previous ward during the education, none had taken a study break. The number of clinical supervisors they had varied from two to getting a new supervisor almost every day. Three students had cared for at least one patient with COVID-19. All students had clinical placements in ICU units where patients with COVID-19 were present (Table 1).

### Data collection

Postgraduate critical care nursing students were recruited during their postgraduate education, initially informed about the study by the heads of both educational programs. If students wanted to learn more and had an interest to participate, they could contact the researchers and receive additional information by letter. After this, a time for interview was set.

**Table 1:** Demographic characteristics of postgraduate critical care nursing students (n = 8)

Variable		Total (n)
Age (years)		26-35
Gender		
	Female	8
	Male	0
Household		
	Living alone without children	1
	Living alone with children	1
	Cohabitating without children	1
	Cohabitating with children	4
	Living with parents	1
Years of experience as RN (years)		4-10
Have a non -ICU specialist education in nursing		1
Have experience as enrolled nurse		4
Nurse trainee		7
Have worked in parallel with the studies		5
Number of supervisors		2-6 (many)
Have taken care of at least one patient with COVID-19		3

Eight out of 44 students participated in individual interviews due to the pandemic performed via Zoom or telephone (four from each university). A study-specific semi-structured interview guide was used, where the open-ended questions focused on learning, supervision, ethically difficult situations, communication with patients and next-of-kin, as well as the impact on their own health during the pandemic. The interview guide was developed by the research group and as such informed by both teachers and clinicians within intensive care. The recorded characteristics of the participants were age, gender, household, experience as a RN, other specialist nursing education, experience as enrolled nurse, nurse trainee, pause during education, regular work, cared for patient with COVID-19 and number of supervisors.

The interviews were performed from March 2021 to August 2021 by three of the authors and lasted between 31 and 54 minutes. Interviews were audio recorded and transcribed verbatim.

The study was conducted in accordance with the Code of Ethics of the Declaration of Helsinki and adhered to the principles of confidentiality, integrity, right to self-determination, and privacy, as well as transparency and secure data processing. The study was given ethical approval by the Swedish Ethical Review Authority. Students received an information letter where the aim and context of the study were described, along with principles of confidentiality and their right to leave the study at any given moment. The researchers who performed the interviews were not involved in any examination or assessment of the students they interviewed.

### Data analysis

The interviews were analyzed by two of the researchers in two steps. First, meaning units containing information about learning were inductively extracted. Thereafter, a deductive approach was used to identify the concepts of attachment, authenticity, autonomy, and professional development as described in a model by Fredholm^13^ depicting the interrelatedness of these concepts. By checking the two individual analysis against each other and discussing themes, some alterations were made for consensus. After the final deductive analysis, all five members of the researcher team read and discussed the themes for consensus regarding the theoretical construct.

In the theoretical model^13^ attachment and authenticity are seen as prerequisites for autonomy in learning. Autonomy in learning is a social phenomenon that evolves in connection to others in situations that are perceived as safe and secure. Autonomy has, apart from independence and control over your own learning, also to do with personal identity and meaning, independent choices, taking responsibility and the ability for critical thinking. Autonomy in learning is closely linked to authentic experiences in clinical education. Authentic experiences can be both external and internal, where external experiences are created by the surrounding environment with real patients in a real clinical setting. Internal authenticity, however, is perceived when students are given the opportunity to form mutual relationships with patients; and when students experience that they contribute in an important way to patient care as part of a team. Authentic experiences, and the perceived meaning of these experiences, can give rise to transformative learning processes that contribute to the development of a professional identity. Both autonomy and authenticity are social phenomena closely connected to the relationships students are allowed to form in a clinical setting. These relationships are a way for students to experience attachment, another vital component in authentic experiences. Attachment is defined as the need to have a sense of belonging in the workplace, with patients, and supervisors, as well as other professional groups, and to the situation and to clinical reasoning.^13^

## Results

**Figure 1:**
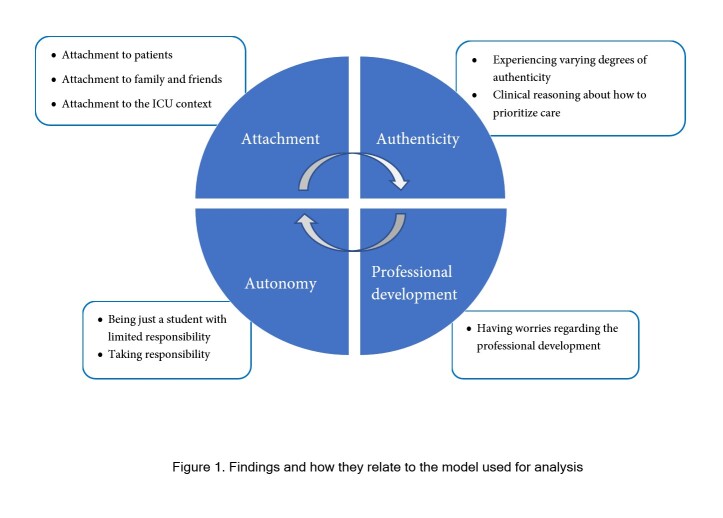
Findings and how they relate to the model used for analysis

When analyzing data using the theoretical model, the main focus of this study was issues related to the concept of attachment (Figure 1). Attachment, or lack thereof, was perceived in relation to patients, family, and friends, as well as to the ICU context. Authenticity was experienced to a varying degree in relation to the reality and demands of COVID-19 care. Autonomy was achieved both voluntarily and involuntarily in relation to responsibility for patients. Issues regarding professional development could also be identified.

### Attachment

Attachment is defined as the need to have a sense of belonging in the workplace, with patients and supervisors, as well as other professional groups, and to the situation and to clinical reasoning.13 Here, this could be seen as a varying degree of attachment to patients, patients’ family, and friends, to the ICU context and to the clinical supervisor.

### Attachment to patients

Students who had the opportunity to care for patients with COVID-19 perceived this as positive. They felt safe and secure in their knowledge about the disease. The pandemic could also be a source of knowledge; and sometimes students perceived that they learned more because of the pandemic and that the care of patients with COVID-19 was rewarding. The care of patients with COVID-19 at later stages in the pandemic could also be described as orderly and offered opportunities for reflection and broadened knowledge about things that would not have occurred otherwise

The use of personal protective equipment (PPE) was experienced to prevent attachment to patients. Students perceived a physical distance to patients, and communication in terms of hearing and talking was negatively affected. PPE had a negative impact on the nurse-patient relationship and made it difficult to console and reassure patients. The use of PPE was sometimes perceived to cause detachment from the patient.

On the other hand, a stronger attachment could also be formed when PPE was used, since you could not enter or leave the room without dressing or undressing.

“But I can really see that you spend more time with the patient as you can’t go back and forth all the time, you can’t take the protection equipment off and on, but you’re there a lot and there is a lot of focus on that patient as you can’t do anything else, and you really want to be there next to the patient even more. (…)” (Student 4: female, 33 years, cohabitating with children, 10 years of experience as RN)

The restrictions regarding visits from family and friends was hard to accept ethically, and the attachment to patients was perceived as dependent on the attachment to friends and family. It contributed in a negative way to an objectification of patients, where the students felt that they were caring for mere bodies in a repeated process without knowing anything about that person’s interests or everyday life. Students found that they talked less to the patients and sometime neglected to inform them about things like different procedures. They worried about patients who had to endure care in the ICU without friends and family and pointed out the particularly stressful environment for patients and the need for support.

“It breaks my heart as a nurse, so to speak. But at my intensive care unit where I am during my work-place practice, I can see that they call as often as possible and let the relatives (family) talk with their loved ones. They have let them talk to each other and when they have been sedated, they have let them hear the voices of their loved ones on speaker, and the rules have been stretched for who to let in. There have been more family members in the room than what has been allowed, as you see that it’s necessary when it’s been a critical disease and stuff and if I compare with what it was like at my other unit where I worked before and the intensive care unit, I almost think that the intensive care unit has been more, you let the patient’s family in a bit earlier, it’s not the last day when they ‘re going to die, but a bit earlier so you can see, and you get the decision-making and so on.” (Student 4: female, 33 years, cohabitating with children, 10 years of experience as RN)

### Attachment to family and friends

Visiting restrictions were imposed early on during the pandemic. These had an impact on the level of attachment that students perceived to patients’ family members and friends. Sometimes, the first encounter with a patient’s loved ones was when they were allowed to visit the patient at the end of life.

“Then of course, we also had family that came in and you were going to end the treatment, and then it feels really hard to enter into that kind of discussion with them. Explain what happens, that you should turn it off and end the life for their loved one, when you wear lots of protective equipment, so then we tried to meet the family just to greet them without all the protection equipment on, and then put it back on again, so you could see each other for a second, and then go on with the conversation. We felt that that was what we could contribute with…” (Student 4: female, 33 years, cohabitating with children, 10 years of experience as RN)

Although the restrictions were perceived as an obstacle, alternative solutions were observed and appreciated by students. Visiting restrictions made family and friends use phones to connect with staff, which sometimes actually improved attachment, due to more frequent and continuous communication.

“Yes, but it was really that yes, family called far more often than what they would have done if they had been allowed to come there, so that it so to speak became a challenge to take the phone calls and just deal with it as good as possible, and yes, in some ways also be there for the patient the way next of kin are, and you had to show that you were there with the patient, like you always do but now even a bit more.” (Student 1: female, 30 years, cohabitating, 5 years of experience as RN)

However, some students felt that they could not form the same bond to family and friends as they normally would and that they were unable to connect on a deeper level and get to know one another.

Regarding student learning, many of the learning goals for the clinical placements entailed learning objectives tied to friends and family. Because of the visitation restrictions, these were difficult to achieve. On the other hand, one student expressed that even if the care was less personal without family and friends, there were some benefits for learning in terms of being able to show insecurities, and it was easier to focus on the patient.

“Well, you do have less background information and it does become less personal when next of kin aren’t there, I think. The only advantage is that if you’re a student, maybe ask a lot and you’re insecure about things to (…) try things out a bit with the patient, but not when family are watching, as you then must really know what you’re doing.” (Student 6: female, 30 years, cohabitating with children, 7 years of experience as RN)

### Attachment to the ICU context

Students expressed a desire to care for patients with COVID-19 in order to learn as much as possible. However, initially during the pandemic it was not possible due to insurance issues. This changed in the spring of 2020. Not being able to care for these patients could leave students feeling like they were missing something important regarding the care of critically ill patients.

Students expressed their compliance to rules and regulations regarding disease control and PPE, thus making it very uncomfortable to watch lower levels of compliance among staff. Procedures and guidelines changed constantly, especially at the start of the pandemic. This was difficult and scary for the students, due to uncertainty among everyone in the unit and due to staff being scared as well. However, it became easier over time as more secure information became available.

“Well, people are scared of course, the staff, there were really a lot of different procedures that were introduced all the time, difficult to get a grip of what it was, how you should do it, I didn’t notice that it should affect my, that it affected my placement in a negative way, no.” (Student 3: female, 30 years, cohabitating with children, 7 years of experience as a RN)

The ICU environment was messy and crowded with staff, patients, and new co-workers in need of introduction. Managers were sometimes perceived as absent, and students felt that they had missed their most important task; to be responsible for the working environment and their staff.

Students perceived that ICU staff had to take charge of their own debriefing, often during coffee or lunch breaks, in the hope that someone would listen to them. Some students had observed a culture in the organisation that did not tolerate criticism and a low interest in the organisation for change or improvement.

The fear of being infected or to infect others was great at the beginning of the pandemic. Although participants mostly reported an unchanged health status, they also found it difficult and stressful to constantly think about COVID-19. This changed somewhat after they were vaccinated. At the beginning of the pandemic, students were more afraid of being infected, which was added to by media reports about infected and dead health care professionals. There was widespread fear that they would bring the corona virus to their homes to infect family and friends. They felt dirty and some were afraid to hug and cuddle with their children. This fear manifested itself even though they intellectually understood that the risk of infection from a mother wearing PPE at work was very small. One student articulated that she could not have handled a loved one becoming infected.

 “I haven’t been afraid for myself. Then you think of your children, but of course, they should not be very affected, but a bit concerned about what you bring home…” (Student 4: female, 33 years, cohabitating with children, 10 years of experience as RN)

### Attachment to the clinical supervisor

The attachment to the clinical supervisor was affected by the supervisor’s and other staff’s discontent with the system due to the pandemic, such as work environment and other working conditions. This could lead to a negative atmosphere and sometimes this affected how students felt they could access the learning environment, ask questions, and participate.

“But one supervisor, I think because of the situation and things like that, she‘s been rather dissatisfied with the organisation itself, so then it was more the dissatisfaction and not the stress, but it was this bit of negative atmosphere, I think it became, God, that I maybe didn’t dare to do as much as when it was more kind of open, so… It was a lot of overtime and no vacation and everything around the pandemic, the organisation, that she kind of… No, not satisfied at all.” (Student 7: female, 26 years, living with parents, 4 years of experience as RN)

In contrast, other students had different experiences, and despite a mood of tiredness and frustration among members of staff, they only had pleasant impressions of everyone, with no negative impact on their clinical placement.

### Authenticity

Authentic experiences can be both external and internal, where external experience is created by the surrounding environment with real patients in a real clinical setting. Internal authenticity, however, is perceived when students are given the opportunity to form mutual relationships with patients and play a real part in the caring process.^13^ Here, this is seen as students wanting to take part in the care of patients with COVID-19, and to take part of clinical reasoning regarding the prioritization of care.

### Experiencing a varying degree of authenticity

The clinical placement turned out to be more or less authentic for the students. It was more narrow and more structured than expected. Due to the pandemic, students had to “go with the flow” and could not get so many of their own wishes regarding learning fulfilled. Some had to change the location of their clinical placement and initially they were not allowed to care for COVID-19 patients.

Some students had the opportunity to care for many severely ill patients in need of mechanical ventilation, something that contributed positively to their learning. The pandemic also meant working under odd and different circumstances, which had a positive impact on learning regarding for instance communication with patients’ friends and family, and also with physicians.

“I believe that the most important thing during the pandemic is that I got a lot of training with really sick patients with hemodynamic impact, so it has been really, really educational. But it has also meant that I have worked during odd conditions, with contact with families and with contact with doctors and yes, everything. Everything has been odd around you. The stage has been different, but the patient clientele you have had to deal with has been very educational. I know that many have thought it’s been hard, but I’ve also seen it as a challenge.” (Student 8: female, 35 years, cohabitating with children, 10 years of experience as a RN)

The students expressed a desire to care for patients with COVID-19 to learn as much as possible and experience the reality of care for this group of patients.

“Yes, but it was really that you were curious about treating COVID patients, that you got to hear a lot from fellow students and colleagues who’d been working at the COVID unit, and that they seemed to be really very sick patients, and you were really eager to care for them, as a student, and there was this sort of feeling that you missed something like, learning opportunities…” (Student 1: female, 30 years, cohabitating, 5 years of experience as RN)

Other students had not cared for as many patients as they had expected and were disappointed that too little happened. Some had to take turns caring for intubated patients when there were not enough intubated non-COVID patients. This had an impact on learning at their clinical placement.

### Clinical reasoning about how to prioritize care

During the extraordinary circumstances with the pandemic, the students had an opportunity to see how patient care was prioritized. How do you prioritize when you suddenly must admit many severely ill patients to the ICU with no beds available?  How do you prioritize among patients where some might not survive? The students had confidence in informed decisions made by physicians. Aware that they were not the ones who needed to make these decisions, students experienced some very difficult situations:

“If someone is in a really bad state, and then you have to say “ok, we might have to intubate you, is there somebody you want me to call?” It has been such an absurd, that was what I was thinking a lot over the past few weeks, as the patients have now, they have now had time to see via media how it might be, that you can die and that it might be the last words you say then, what do you want to say before we get you intubated? It is a horrible kind of thing, and especially last week, then it was one who said “no, I can’t take it anymore, I can’t take it”. “Ok, but I can call somebody for you, but who do you want me to call?” That you must make these decisions when you are so damned sick (…) (Student 5: female, 34 years, living alone without children, 7 years of experience as a RN)

The students also experienced discussions about when family members should be allowed come and visit.

“One really has to decide; how sick is this patient and when should we call in their next of kin, because you can’t do it too early, and you don’t want to do it too late. Usually, you might not have had to think so much about it (…). (Student 5: female, 34 years, living alone without children, 7 years of experience as a RN)

### Autonomy

Autonomy in learning is a social phenomenon that evolves in connection to others in situations that are perceived as safe and secure. Autonomy has, apart from independence and control over your learning, also to do with personal identity and meaning, independent choices, taking responsibility and having an ability to think critically.13 Here, this is seen as the need for different degrees of responsibility in relation to the degree of support from the clinical supervisor.

### Being just a student – with limited responsibility

The students did not want to be fully responsible for patients all the time; they expressed a need for improvisation and to be able to do whatever they thought they needed to learn more about.

“So, you don’t kind of feel that you have to be fully responsible for the patient, if I need, I can step aside, read up on a treatment, an examination or whatever it can be and then if something else happens at the unit that might be a bit unusual (…) a chance to go there and see it, that I don’t need to feel that I have to be with the patient just because I sat there for that day.” (Student 4: female, 33 years, cohabitating with children, 10 years of experience as RN)

By just being a student, they had the opportunity to observe the team, which reduced the pressure of being in charge of patients.

“Well, it’s been so damned fun, or it’s always fun to be a student, because you can observe, I love to observe teams at work. I enter a context and “just being a student” I can really just stand there, you don’t have the pressure to be responsible for the patients and that, so then I think it’s been a lot of fun to observe how it works with hierarchy and so on, how they work together, it’s been a lot of fun.” (Student 5: female, 34 years, living alone without children, 7 years of experience as a RN)

### Taking responsibility

After a while, students felt that they could gradually take over responsibility and be in charge of a patient.  It was possible with support from supervisors, with individual adjustments for each student.

Opportunities for reflection with the supervisors varied. Some felt that they had had opportunities to reflect continuously during the work shifts, for example in connection with a blood gas or documentation. Others felt that it was not possible during every work shift. The students often initiated the need for reflection and the supervisors managed to find time and space for it.

“…that I’m allowed to try first for myself and think and find the answer on my own, instead of giving me the answer, and it’s also that you need time for reflection, to stand and think, and that it might take a bit longer, but that you at least have the opportunity to reach the answer yourself, so then I kind of want to get in there and do everything with the patient, all the routine and kind of start the day on my own and maybe be very independent during the round, that I get the chance to say what I know, if it might be that I have forgotten something, the supervisor can jump in, but that no one takes over the situation.” (Student 4: female, 33 years, cohabitating with children, 10 years of experience as RN)

The students felt they had developed their skills despite the exceptional circumstances of the pandemic, thanks to the supervisor’s efforts and support. The pandemic and the extreme situation could even be regarded as offering an unusual opportunity for development.

“…how they manage to be so nice and educational despite having such a large workload and burden of care, that’s amazing, and still kind of take time for me as a student and let me feel important and not in the way, I think it’s been very good in that way. And you’ve had the opportunity to develop in a way I believe you wouldn’t have done if it hadn’t been for this exceptional situation.” (Student 2: female, 32 years, recently divorced, cohabitating with children, 5 years of experience as RN)

A student with new supervisors almost every day experienced that her learning curve was not as good as expected, but when the supervisors gained trust in her, she could care for the patients more independently:

“… and I had really wished to have a more coherent supervision so that I didn’t have to start all over again every day, as my development curve wouldn’t have become as steep as it had been maybe, if I had had a person who knows me and know what I can and cannot do. Now every day I had to start all over again and explain what I can and cannot do and that they can feel safe with me and so on, and hope that they relax their control over me, even though I’m on my last week (…). But it has gone surprisingly well, it has been, they have relaxed their need for control, and I’ve been allowed to be quite independent (…)  which I felt that I wanted.” (Student 4: female, 33 years, cohabitating with children, 10 years of experience as RN)

The students also felt that they could care for the patients more independently when the supervisors had other commitments – with a temporary supervisor nearby. For a short while, they felt like ICU nurses.

“I’ve never been in that situation like, you can take this patient on your own because I have to take care of this one as we are too short on staff, I don’t think it’s been that way. But it has been more of, one of my supervisors is working for the union, so she sometimes went away on meetings (…) things and replacements and stuff like that, and then you were on your own for maybe an hour, but there is always someone to ask.” (Student 6: female, 30 years, cohabitating with children, 7 years of experience as a RN)

There were sometimes situations where the students felt that they were given nearly too much responsibility. For instance, when it came to new staff, some who did not work in the ICU, but were involved in the care of patients with COVID-19 as well as patients with other diagnoses.  There had been situations when their supervisors were busy elsewhere when the students were the ones who were the most experienced in intensive care compared to the new staff.

“What I felt, I hoped that somebody would get in there quickly, that the patient wouldn’t become even more sick, because it was me, it still felt like I had the responsibility because the person who was there to relieve, she doesn’t have control, I was becoming a bit stressed, but luckily it was stable. My supervisor came in quickly, or an assistant nurse.” (Student 7: female, 26 years, living with parents, 4 years of experience as a RN)

### Having worries regarding one’s professional development

Experiences of authenticity and autonomy, and their perceived meaning, can create transformative learning processes that contribute to the development of a professional identity.^13^

The students thought it would be exciting to soon be able to work as ICU nurses, but also a bit frightening. Being a student means lower expectations; you can ask as many questions as you want to, there is a more permissive attitude towards that during the clinical placement. When you have passed your examination, the ICU staff will expect you to be more independent.

“Not being a student, but now I will have the role and be independent and take care of patients, and make decisions, and that is the independent role, yes. But they’re really so sick so you have to react quickly too, yes, I guess that’s what’s scary.” (Student 7: female, 26 years, living with parents, 4 years of experience as a RN)

They had experienced a rather harsh jargon in the staff room when the nursing staff talked about their coworkers. For example, questioning whether someone was competent to do something, or discussing that someone took too long to perform a certain task. The students wondered if that was how the ICU staff would talk about them when they returned as fully trained ICU nurses. What if they were not good enough?

“…imagine I don’t have anything, imagine I cause annoyance among colleagues, that yes, God, she’s so difficult to work with (…) she’s new, and she asks a lot of questions, and she’s not as independent as she should be, and she ought to know this. I have lots of difficult thoughts like that, and I guess a kind of performance anxiety, and I get that very easily, and when it comes to this work, you can’t do that, but it always has to be done right, and then it feels difficult.” (Student 2: female, recently divorced, cohabitating with children, 5 years of experience as a RN)

Furthermore, they expressed concerns about ICU nurses who quit and the loss of competence and experience this brought. What will it be like to work as an ICU nurse? Are you allowed to be new at work? As a new ICU nurse, will you get the support you need? Maybe you would like to quit rather than continue as an ICU nurse.

“I do feel that I’m afraid that I’m not going to like this work in the end, because it’ll be too much and too hard.” (Student 3: female, 33 years, cohabitating with children, 7 years of experience as a RN)

The lack of experience in caring for patients with COVID-19 that some of them had was a disappointment and they expressed concerns about not having this specific competence. Therefore, they were very keen to start caring for those patients as soon as possible.

## Discussion

The aim of this study was to explore intensive care nursing students’ experiences of learning in the ICU during the COVID-19 pandemic, and to understand these experiences in relation to self-directed learning and professional development. The students all expected and felt that they received the best possible supervision. Given the circumstances, generally had a positive experience. Students did not want to be a burden to supervisors but wanted to contribute to patient care. This caused them to sometimes be reluctant to ask questions and they found it difficult to make their supervisor slow down. Students were impressed with the CCN:s managing to be friendly and pedagogical despite the extra workload and strain on them. Nurses were competent and secure in their professional roles, but students sensed that supervisors felt stress and inadequacy due to the COVID-19 pandemic. Sometimes students were forced to take on more responsibility to help staff, which the students perceived as something positive. Other times, they had to take a step back when supervisors had a lot to do, something they perceived as negative.

When viewing findings deductively through the lens of attachment, authenticity, and autonomy, we can see that issues related to attachment are more dominant. Studies about belonging and belongingness (the need to be involved with

others, being part of, feeling accepted and fitting in, being cared about, valued, and respected)^1^^,^^6^ show similar findings, but it is important to note that these studies do not include specialist nursing students. Which role does attachment play? And are there special issues relating to the pandemic? Or is the focus on attachment a result of these students already having a professional identity as nurses, and only now need to belong in an ICU context? Legitimate peripheral participation as described by Lave & Wenger^23^ is a way for students to take active part in practice and to experience the physical environment, the affordances, activities, language, artefacts, and so on, within a community of practice without demands on full participation.

Two factors were experienced as particularly affecting attachment: the PPE and the visitation restraints. The use of PPE often had a negative impact on the students’ communication and therefore attachment with patients, staff, and patients’ family members. Andersson and colleagues^20^ found that PPE could enhance nurses’ experiences of moral distress as the equipment made it difficult to communicate, reducing communication and interaction with patients and colleagues to a minimum.

A recent study by Wendlandt and colleagues^24^ found both positive and negative effects of visiting restraints in the ICU during the COVID-19 pandemic. Both nurses and physicians were concerned about an overall negative impact on patients and their loved ones. However, while nurses noted “positive changes to daily workflow and ability to provide medical care in the absence of family visitors, driven by increased time and physical space to provide direct patient care”, “physicians reported negative changes to daily workflow and ability to provide medical care in the absence of family visitors, driven by disruptions in family communication”. Andersson and colleagues^20^ describe how the visiting restraints made it difficult for CCN:s to get to know the patient as a person, since a critically ill person seldom can express his or her personal needs, resources, and interests – thereby making the ill person become just another COVID-19 patient among others.

The students expressed concerns about transmitting the COVID-19 infection to their families, concerns they shared with ICU staff around the world.^25^ They noticed that some of their friends withdrew, a finding in line with Gordon and colleagues^26^ who found that ICU nurses endured “stigmatization attitudes by those viewing them as virus carriers”. Andersson et al.^20^ also found that CCN:s in the first phase of the COVID-19 pandemic, feared becoming infected and spreading the infection to others.

The students in this study all experienced a varying degree of authenticity, and those students who did not have the opportunity to care for patients with COVID-19 experienced authenticity to a lesser degree. At the same time as authenticity was desired, they were also relieved that they were sometimes allowed to just be a student with limited responsibility and still have their experience as an RN. Sometimes, the conditions of the care with limited resources, staff, and supervisors, made students take on responsibility that they normally would not have had the opportunity to do. This was mostly appreciated but could also result in feelings of insecurity and dread. Another source of authenticity was their participation in discussions and clinical reasoning regarding how to prioritize care, something that felt difficult and foreign to students. According to Lave & Wenger^23^ newcomers must have broad access to arenas of a mature practice, but at the same time productive peripheral participation requires less demands on time, effort, and responsibility, than for full participants.

In general, the students in this study were content with their learning during the clinical placement, however, our results showed that students were concerned about whether their professional development was good enough and whether they had gained the proper competence. Furthermore, they raised worries about the competence gap in ICUs that is growing, as CCN:s were quitting their jobs due to the pandemic. Newly trained ICU nurses will be introducing and supervising new colleagues, as well as specialist nursing students, a responsibility that might be difficult to bear.^20^ Transparent and timely leadership communication correlates to decreased moral distress.^27^ Another successful strategy is team discussions, in a structured and guided debriefing format, so that each CCN will have the opportunity to reflect on their feelings and coping strategies with others.^28^ Forming supportive relationships is another important strategy, along with peer support and facilitation of socialization in the ICU context.^29^ These strategies should be available for students as well. Lave & Wenger^23^ claim that identity development is central to the careers of newcomers in communities of practice. Thus, participation in a community of practice is more than a process of learning, but a reciprocal relation between person and practice. The move towards full participation does not take place in a static context, and it changes over time and circumstances. Our interpretation, in the light of our findings, is that there is a need for attachment and that students need an opportunity to form this attachment.

### Limitations

There are some limitations to this study. The sample was a convenience sample of students from two universities and the results may not be transferrable to other universities. The sample size is small even for a qualitative study. However, the richness of data and the following theoretical analysis conducted by multiple researchers can be seen as enhancing trustworthiness and transferability. Also, the similarities between the students’ narratives in the interviews are contributing to trustworthiness. The deductive approach in the analysis can be viewed as both an enrichment and a limitation. The strength in the approach lies in the enhanced transferability, and the weakness in the possible risk of missing important information. This risk, however, is reduced through multiple readings of the texts and a constant going back and forth to check the parts against the whole.

## Conclusions

The concepts of attachment, authenticity, and autonomy are relevant and useful for examining students’ experiences of clinical practice. However, in this study, attachment comes to the fore as a concept of importance for postgraduate critical care nursing students in training, possibly indicating that for them as RN's, the new context was perceived as the main learning object. Findings show the need for participation in the ICU community of practice without the demands and responsibility of full participation. To learn how to become a critical care nurse, students need to be given the opportunity to form a relationship with the practice or context, making this an important learning objective. For attachment, participation, and consequently professional development to take place, students need to be invited to be a part of the team even during such straining circumstances as an ongoing pandemic. For this to become a reality, both the clinical practice and the university need to be aware of the importance of the authentic involvement of students. These findings can advance the understanding of how to organize clinical education during future crises such as a new pandemic. Future studies should focus on strategies and interventions for making students more involved.

### Conflict of Interest

The authors declare that they have no conflict of interest.
